# Identification of novel modulators of a schistosome transient receptor potential channel targeted by praziquantel

**DOI:** 10.1371/journal.pntd.0009898

**Published:** 2021-11-03

**Authors:** Evgeny G. Chulkov, Emery Smith, Claudia M. Rohr, Nawal A. Yahya, Sang-Kyu Park, Louis Scampavia, Timothy P. Spicer, Jonathan S. Marchant

**Affiliations:** 1 Department of Cell Biology, Neurobiology and Anatomy, Medical College of Wisconsin, Milwaukee, Wisconsin, United States of America; 2 Department of Molecular Medicine, Scripps Research, Jupiter, Florida, United States of America; 3 Department of Pharmacology, University of Minnesota Medical School, Minneapolis, Minnesota, United States of America; University of Pennsylvania, UNITED STATES

## Abstract

Given the worldwide burden of neglected tropical diseases, there is ongoing need to develop novel anthelmintic agents to strengthen the pipeline of drugs to combat these burdensome infections. Many diseases caused by parasitic flatworms are treated using the anthelmintic drug praziquantel (PZQ), employed for decades as the key clinical agent to treat schistosomiasis. PZQ activates a flatworm transient receptor potential (TRP) channel within the melastatin family (TRPM_PZQ_) to mediate sustained Ca^2+^ influx and worm paralysis. As a druggable target present in many parasitic flatworms, TRPM_PZQ_ is a promising target for a target-based screening campaign with the goal of discovering novel regulators of this channel complex. Here, we have optimized methods to miniaturize a Ca^2+^-based reporter assay for *Schistosoma mansoni* TRPM_PZQ_ (*Sm*.TRPM_PZQ_) activity enabling a high throughput screening (HTS) approach. This methodology will enable further HTS efforts against *Sm*.TRPM_PZQ_ as well as other flatworm ion channels. A pilot screen of ~16,000 compounds yielded a novel activator of *Sm*.TRPM_PZQ_, and numerous potential blockers. The new activator of *Sm*.TRPM_PZQ_ represented a distinct chemotype to PZQ, but is a known chemical entity previously identified by phenotypic screening. The fact that a compound prioritized from a phenotypic screening campaign is revealed to act, like PZQ, as an *Sm*.TRPM_PZQ_ agonist underscores the validity of TRPM_PZQ_ as a druggable target for antischistosomal ligands.

## Author summary

The drug praziquantel is used to treat diseases caused by parasitic flatworms. Praziquantel is an old drug, and there is a need to identify novel treatments that retain desirable features and improve weaknesses in the mode of PZQ action. One way to do this is to identify new drugs that exploit vulnerabilities in the same drug target but work in slightly differently ways. Here, we have optimized high throughput screening methods to pharmacologically profile a parasitic flatworm ion channel targeted by PZQ. We have identified several new chemical structures that interact with this channel complex. These ligands provide new opportunity for developing tools to manipulate flatworm biology and potentially new trajectories for anthelmintic drug development.

## Introduction

Over a billion people worldwide require chemotherapy for neglected tropical diseases (NTDs, [1]). Schistosomiasis, a disease caused by infection by parasitic flatworms known as schistosomes, is one of several NTDs targeted for elimination as a public health problem in the World Health Organization 2021–2030 NTD road map [1]. Schistosomiasis, as well as several other parasitic flatworm infections [2], are treated using the anthelmintic drug praziquantel (PZQ). PZQ has remained an effective treatment for schistosomiasis over four decades of clinical use [3], underpinning recent mass drug administration (MDA) campaigns aimed at decreasing infections and morbidity in vulnerable populations. Alternatives to PZQ are however needed. PZQ has several features that could be improved and the threat of drug resistance, potentially accelerated by the rollout of MDA initiatives, persists [4–6].

We recently discovered that PZQ activates a Ca^2+^-permeable ion channel in *Schistosoma mansoni* that belongs to the melastatin family of transient receptor potential (TRP) channels (christened *Sm*.TRPM_PZQ_ [7,8]). PZQ also acts a potent activator of TRPM_PZQ_ in other PZQ-sensitive parasites [9]. TRP channels, which act as non-selective cation channels, are appealing targets for anthelmintic drug discovery owing both to their important physiological roles in sensory physiology as well as their druggability [10–15]. However, little is currently known about the pharmacology of flatworm TRP channels. Efforts to profile these channels will be important for validating tools to selectively manipulate worm physiology, as well as for anthelmintic development [13–15]. Insight to date suggests schistosome TRP channels, like other flatworm targets [16], exhibit a different pharmacological profile compared with their closest mammalian counterparts. For example, *Schistosoma mansoni* TRPA1 responds to capsaicin, a human TRPV ligand [17]. *Sm*.TRPM_PZQ_, which harbors a TRPM8-like binding pocket in the voltage-sensor like domain (transmembrane helices S1-S4, [9]), is not activated by the human TRPM8 agonists menthol and icilin [7]. Customization of these TRP channels to parasite-specific functions may underpin this divergence and specialization.

To characterize flatworm TRP channel pharmacology, it would be helpful to establish methods for screening individual channels against diverse drug libraries. *Sm*.TRPM_PZQ_ is a good candidate for optimizing such an approach given it is a targeted by PZQ, and mediates a large, sustained Ca^2+^ signal in heterologously expressing cells. In collaboration with the Molecular Screening Center at Scripps Research in Florida, we optimized target-based screening approaches for *Sm*.TRPM_PZQ_ with the goal of discovering other ligands that engage this ion channel complex [18]. The hope is that by optimizing high-throughput screening (HTS) methods for TRPM_PZQ_, new chemotypes distinct from PZQ can be identified including ligands that act at different sites on the channel relative to the transmembrane PZQ-binding pocket [9]. These could include allosteric modulators, or ligands that interact with the pore-forming domain (S5-S6). Such ‘hits’ could then be further iterated and evaluated as leads for anthelmintic development.

Here, we executed a pilot screen of ~16,000 compounds against *Sm*.TRPM_PZQ_ which identified numerous antagonists as well as a single novel activator of *Sm*.TRPM_PZQ_. Interestingly this *Sm*.TRPM_PZQ_ activator is a known chemical entity previously prioritized from a phenotypic screen of schistosome worms. These data underscore first, the feasibility of a high throughput screening (HTS) approach for flatworm TRP channels, and second convergence of target- and phenotype-based screening approaches on the same ligand here revealed to act as a *Sm*.TRPM_PZQ_ agonist.

## Results

**Fig 1:**
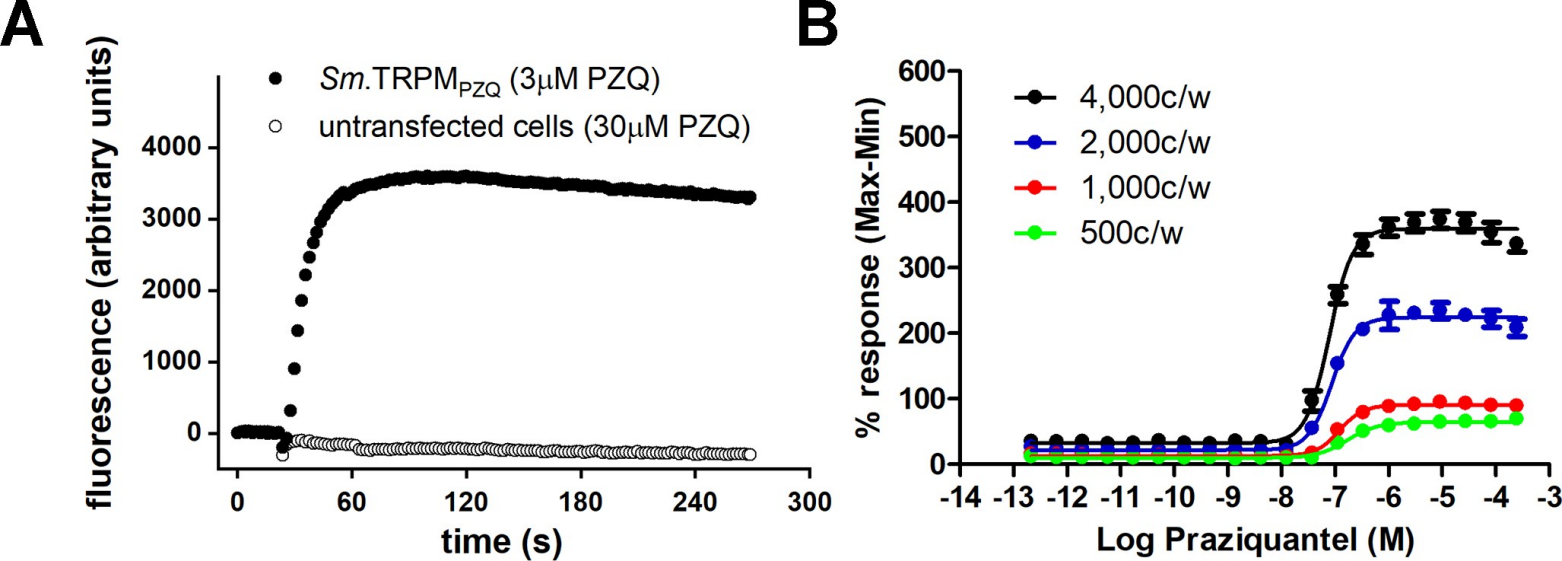
Optimization of assay conditions for monitoring *Sm*.TRPM_PZQ_ activity. (**A**) Representative fluorescence trace showing the kinetics of the Ca^2+^ signal resulting from addition of PZQ (3 μM) to HEK293 cells transiently expressing *Sm*.TRPM_PZQ_ (closed symbols), or addition of a higher concentration of PZQ (30 μM) to untransfected HEK293 cells (open symbols). (**B**) Concentration response curves of PZQ-stimulated Ca^2+^ signals in *Sm*.TRPM_PZQ_ expressing HEK293 cells resolved at various densities of cells per well (c/w) measured in suspension in 1,536 well plates at room temperature. Each point on each curve represents the average of responses from 16 replicates which were also run in 2 separate experiments to achieve average and standard deviation.

Activation of *Sm*.TRPM_PZQ_ heterologously expressed in HEK293 cells was resolved by following changes in fluorescence emission of a synthetic Ca^2+^ indicator over time [7]. In cells, transiently expressing *Sm*.TRPM_PZQ_ and seeded into a 96-well plate, addition of ±PZQ (3 μM) caused a rapid, dose-dependent increase in fluorescence (Fig 1A, [7]). Addition of higher concentrations of PZQ (≤30 μM) to untransfected HEK293 cells failed to elicit any change in basal fluorescence (Fig 1A, [7]).

In order to support a large scale HTS, we trialed various conditions to support miniaturization of this basic reporter assay into smaller volumes necessary for screening in 1536-well format. Miniaturization of the screening assay was aided by the large amplitude of the *Sm*.TRPM_PZQ_–dependent Ca^2+^ transient (change of fluorescence/basal fluorescence, ΔF/F = 12.3±2.1 for PZQ signals at *Sm*.TRPM_PZQ_ versus ΔF/F = 4.5±1.5 for ATP signals through endogenous receptors as measured by confocal Ca^2+^ imaging [7]) and the non-desensitizing nature of the PZQ-evoked Ca^2+^ signal, which was sustained over several minutes (Fig 1A). Experimental performance was compared (i) using various densities of *Sm*.TRPM_PZQ_-transfected cells assayed in suspension, (ii) at different temperatures (room temperature (RT) versus 37°C), and (iii) using either freshly transfected cells, or thawed stocks of previously frozen transfected cells. Each condition was trialed in a 1,536 well format using the fluorescent dye calcium-5 (K_d_ for Ca^2+^ = 390 nM). Assay performance under these screening conditions was compared by calculating the Z’ factor (Z’), a widely used indicator of HTS assay robustness [19], as well as by monitoring the dynamic range of the assay (signal[F_max_]:basal[F_basal_], S:B). Z’ values over 0.5 are considered to be a prerequisite for an excellent HTS.

Assays in the 1536 well format demonstrated that increasing the cell count per well resulted in increased signal and dynamic range (Fig 1B), as well as a decrease in the measured EC_50_ (from 135±15 nM at 1000 cells/well, to 79±2 nM at 4000 cells/well, Table 1). The temperature at which responses were recorded (RT versus 37°C) did not change the sensitivity of *Sm*.TRPM_PZQ_ under these assay conditions (Table 1). Optimal conditions for executing the assay were selected as 4000 cells/well in suspension at room temperature, where assay performance exceeded needed parameters (Table 1). Similar assay performance was achieved using either fresh cells (EC_50_ = 79±2 nM, Z’ = 0.82±0.03, S:B = 10.05±0.08, Table 1) or thawed cells from previously frozen stocks (EC_50_ = 90±10 nM, Z’ = 0.86±0.02, S:B = 10.55±0.08) under the same assay conditions. Therefore, to further minimize assay variation, pilot screens were all performed using a single batch of transfected cells prepared in bulk and then frozen. This facilitated execution of screens and removed transfection efficiency as an experimental variable. Data from all of these trials are summarized in Table 1.

**Table 1:** Assay metrics under specified conditions. EC_50_, Z’ and S:B were determined for *Sm*.TRPM_PZQ_ activation by PZQ at indicated cell densities and temperatures. Conditions in bold text indicate the final conditions selected for the screen. For each condition, responses were found to be positively cooperative (Hill coefficients range of 1.5–2).

Cell density	Temperature	EC_50_ (nM)	Z’	S:B
500 c/w	RT	195±25	0.31±0.37	5.55±0.73
500 c/w	37°C	202±4	0.44±0.14	7.28±0.28
1000 c/w	RT	135±15	0.48±0.07	5.73±0.06
1000 c/w	37°C	144±2	0.53±0.08	7.41±0.01
2000 c/w	RT	95±7	0.69±0.04	8.23±0.17
2000 c/w	37°C	108±2	0.79±0.03	8.04±0.39
**4000 c/w**	**RT**	**79±2**	**0.82±0.03**	**10.05±0.08**
4000 c/w	37°C	80±1	0.83±0.01	9.24±1.23

**Fig 2:**
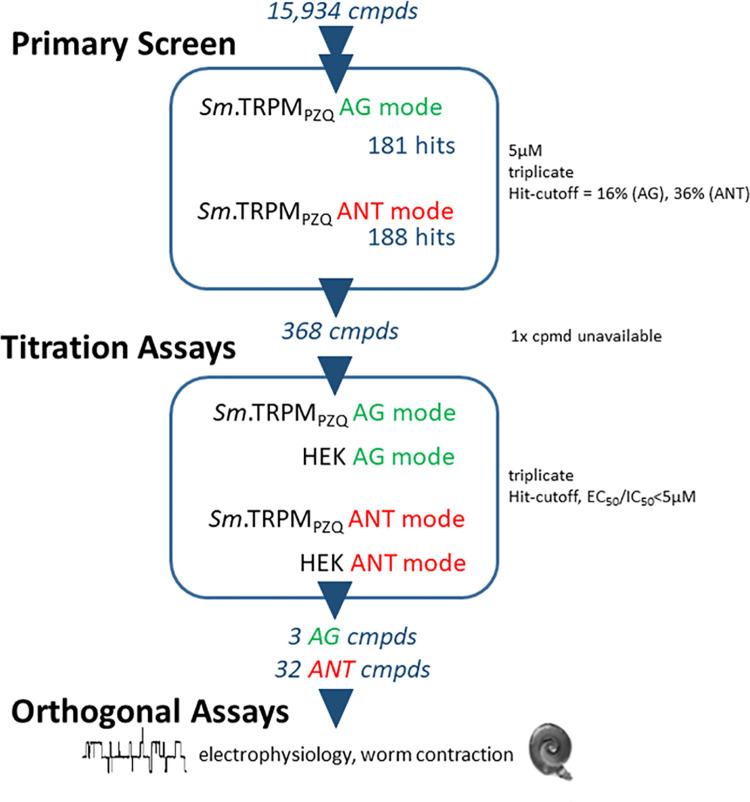
*Sm*.TRPM_PZQ_ screening pipeline. All HTS assays were performed in 1,536 well format. A fixed concentration (nominally 5 μM) primary screen was followed by titration assays and counter-screening in untransfected HEK cells. All assays were performed in triplicate in both agonist (AG) and antagonist (ANT) mode. From the original 15,934 compounds (cmpds) screened, the pipeline yielded 3 putative agonist ‘hits’ (two hits were identified as PZQ present as a ligand in the screened libraries) and 32 putative antagonist ‘hits’. Selected compounds were validated using electrophysiology and by monitoring contraction of schistosome worms *ex vivo*.

**Fig 3:**
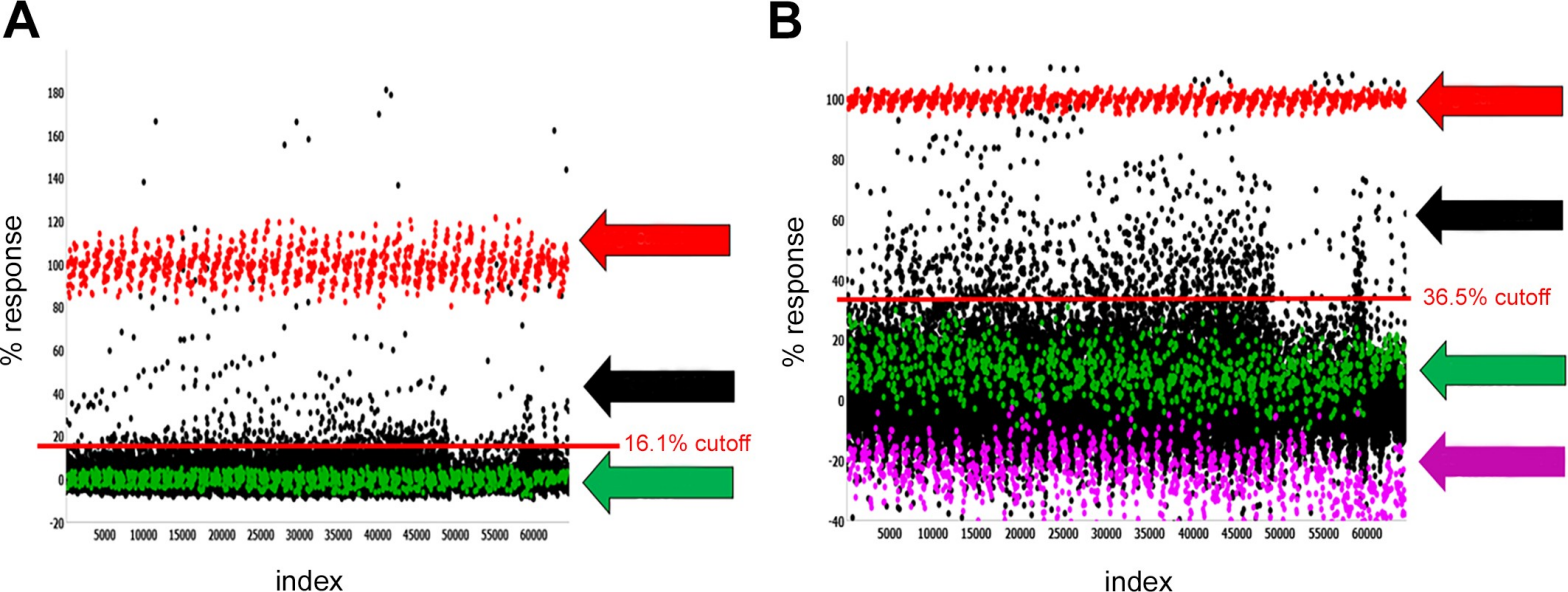
Primary HTS assay data. Scatterplot of all 15,934 compounds tested in (**A**) agonist mode and (**B**) antagonist mode. Each dot graphed represents the activity of a well containing test compound (black, sample field) or controls (red high control; green, low control). Arrows (right) indicated the high control (red), low control (green) and the sample field (black, datapoints between the calculated cutoffs (red lines) and high control). For the agonist screen, high control reflected responses to PZQ (10 μM, red) and the low control represented responses to DMSO (green). For the antagonist screen, the high control reflected responses to DMSO (red) and the low control represented responses to submaximal PZQ (500 nM, green). The response to a maximal PZQ concentration (EC_MAX_, 10 μM) is also indicated (magenta). The EC_80_ stimulation is qualified as a percentage of the response to the high control PZQ EC_100_ (‘EC_MAX_’). All plates are assessed for robustness with Z’ >0.5 and EC_Stim_ between 70–95%.

After optimizing conditions for miniaturization into a 1,536 well format, we proceeded to execute a screening campaign against a total of 15,984 compounds. The screened libraries comprised LOPAC_1280_ (1280 compounds), the Pathogen Box (400 compounds) and a subset of the in-house Scripps Drug Discovery Library, which included the Maybridge Hitfinder library. The screening pipeline consisted of (i) a primary, fixed concentration screen (5 μM) performed in triplicate in both ‘agonist’ and ‘antagonist’ mode, followed by (ii) titration and finally (iii) counter-screening assays of all putative ‘hits’. For the primary screen, following compound addition and completion of the ‘agonist’ (AG) mode read of 3 minutes, the ‘antagonist’ (ANT) mode commenced by PZQ addition (at an EC_80_ concentration) to wells that contain either compounds or DMSO (‘low control’). The output was compared to the response of wells with DMSO without PZQ stimulation (‘high control’). A summary of the workflow and observed compound attrition through each of these steps is shown schematically in Fig 2. Single point scatterplots from all compounds tested in the primary screen are shown in Fig 3.

Assay performance in the primary agonist screen met quality thresholds (Z’ = 0.71±0.04, S:B = 11.40±0.04) and the observed sensitivity to PZQ was consistent with the prior assay optimization trials (EC_50_ for PZQ = 101±3 nM). Assay performance in the primary antagonist screen also met quality thresholds (Z’ = 0.72±0.04, S:B = 9.4±0.4). Hit cut-offs for both screening modes was determined using an interval-based algorithm [20,21]. The sample field in the agonist screen was taken as a percentage response (‘hit-cutoff’ >16%) which identified 181 putative ‘hits’ for further progression. The initial sample field in the antagonist screen was calculated as a ‘hit-cutoff’ >36%, which identified 188 putative ‘hits’ for further evaluation.

A total of 368 compounds were then advanced to titration screening from the primary screen ‘agonist’ and ‘antagonist’ assays (1 compound was not commercially available). Each of these 368 compounds was profiled as a 10-point dose-response analysis run in triplicate against untransfected HEK cells (‘counterscreen’) as well as HEK cells expressing *Sm*.TRPM_PZQ_. Assay performance in these titration assays met required specifications (S1 Table). After curve-fitting, compounds displaying an EC_50_>5 μM (agonist) or IC_50_ >5 μM (antagonist) were considered ‘inactive’ and not studied further. The surviving ‘hits’ that progressed through this pipeline comprised 3 potential agonists and 32 potential antagonists. Full details of these hits and associated assay data are provided in S2 and S3 Tables.

**Fig 4:**
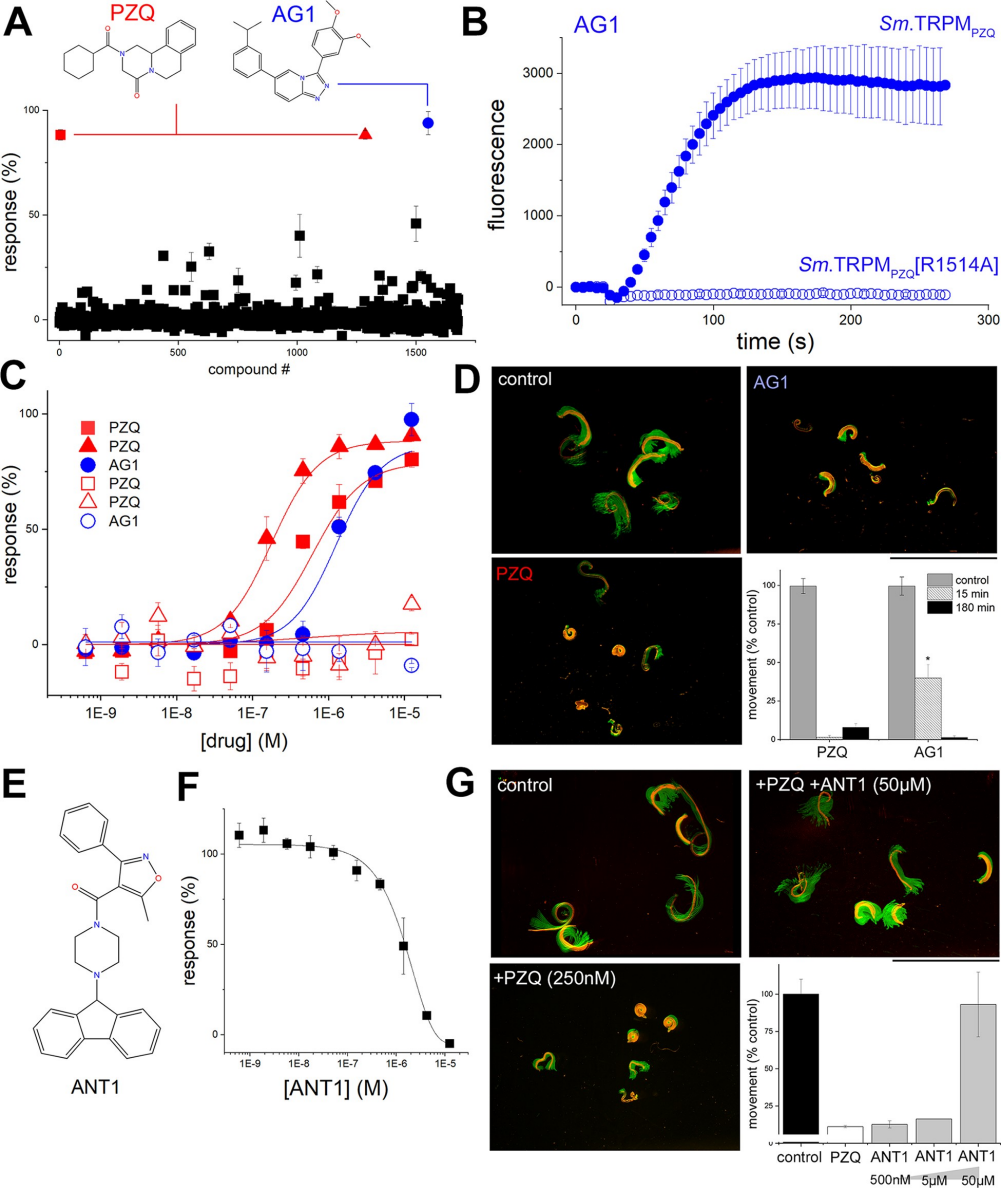
Identification of new chemotypes active at *Sm*.TRPM_PZQ_. (**A**) Primary screen of *Sm*.TRPM_PZQ_ in agonist mode measuring peak Ca^2+^ signal amplitude in response to 1,678 compounds (LOPAC_1280_, Pathogen Box) tested at 5μM final concentration. Structures of ‘hits’ (PZQ, red; AG1, blue) are shown. (**B**) Kinetics of a response of wild-type *Sm*.TRPM_PZQ_ to AG1 (30μM, closed circles), or the binding pocket mutant *Sm*.TRPM_PZQ_[R1514A] to AG1 (open circles). (**C**) Analysis of the three primary screen hits via full concentration response curves for PZQ (compounds #5, #1287) and AG1 (compound #1552) in HEK293 cells transiently transfected with *Sm*.TRPM_PZQ_ (solid symbols) or untransfected controls (open). Data represent mean±sd of triplicate samples. (**D**) Images of adult schistosome worms with single frame image (red) overlayed with maximum intensity projection (green) of a time-lapse series to illustrate worm movement and effects of PZQ (500 nM) and AG1 (1 μM) on worm motion 15 mins after treatment. Graph shows effects of drugs on worm mobility after a 15 min and 180 min exposure (*, p<0.01). Data are analyzed from n≥3 independent infections. (**E**) Structure of a putative *Sm*.TRPM_PZQ_ blocker (ANT1) from HTS screening activities. (**F**) Concentration-dependent blockade of *Sm*.TRPM_PZQ_ dependent Ca^2+^ signals (evoked by 500 nM PZQ) by increasing concentrations of ANT1. (**G**) Images of adult schistosome worms with single frame image (red) overlayed with maximum intensity projection of a time-lapse series to illustrate worm movement (green) and the effect of PZQ (250 nM) as well as PZQ in the presence of ANT1 (50μM). Data are captured after 24 hours of incubation. *Inset*, quantitative analysis of data from n = 3 independent infections.

All three agonist hits were evaluated in further detail. Two of the agonist hits were identified from the Pathogen Box, and one from LOPAC1280. The primary screening data and titration analysis from the plates containing these compounds were extracted from the screening dataset. Two of these hits were identified as PZQ (red symbols, Fig 4A) as both the Pathogen Box and LOPAC1280 libraries contained PZQ as a test ligand. A third agonist ‘hit’ (christened ‘AG1’, blue circle), from the Pathogen Box, elicited strong activation of *Sm*.TRPM_PZQ_ (B_max_ = 93.9±5.6%) in the primary screen (Fig 4A). AG1 is a known chemical entity (3-(3,4-diimethoxyphenyl)-6-(3-(propan-2-yl)phenyl)-[1,2,4]triazolo[4,3-a]pyridine, MMV688313; Fig 4A) and represents a chemotype distinct from PZQ. Addition of AG1 (100 μM) resulted in a sustained cytoplasmic Ca^2+^ signal in cells expressing *Sm*.TRPM_PZQ_ resembling the action of PZQ (compare Fig 4B with Fig 1A). AG1 action was also assessed against a *Sm*.TRPM_PZQ_ channel mutant (*Sm*.TRPM_PZQ_[R1514A]) in the fourth transmembrane spanning helix (TM4) that blocks PZQ action by ablating interactions necessary to shape the PZQ binding pocket [9]. As expected, PZQ did not elevate Ca^2+^ in cells expressing *Sm*.TRPM_PZQ_[R1514A] (Fig 4B). However, this binding site mutant also ablated AG1 activity (Fig 4B), suggesting that AG1 also acts as an orthosteric ligand. Concentration-response curve analysis revealed AG1 acted as a full agonist of *Sm*.TRPM_PZQ_ (EC_50_ = 1.6±0.3 μM) in Ca^2+^ flux assays, with no activity observed on counter-screening in naïve HEK cells (Fig 4C). The potency of AG1 was lower than observed with either sample of PZQ (EC_50_s of 177±21 nM, 619±225 nM) present in two libraries screened under identical conditions (Fig 4C). Both ligands were then re-sourced for validation assays and activities were re-assessed from independently procured samples (PZQ, EC_50_ = 406 nM; AG1, EC_50_ = 9.2 μM). Finally, the action of AG1 was studied against adult schistosome worms isolated from infected mice. Addition of AG1 to schistosome worms *ex vivo* evoked a sustained contraction (Fig 4D), although the kinetics of onset of the AG1-evoked contraction were slower than observed with PZQ. Collectively, these data identify AG1, a distinct chemical entity from PZQ, as a novel *Sm*.TRPM_PZQ_ agonist.

Screening in antagonist mode identified several 32 potential blockers of *Sm*.TRPM_PZQ_ (S2 Table). To begin analysis of this larger set of ligands, we investigated that action of one of the more potent blockers, the compound ANT1 (1-(9H-fluoren-9-yl)-4-(5-methyl-3-phenyl-1,2-oxazole-4-carbonyl)piperazine, Fig 4E). ANT1 blocked PZQ-evoked Ca^2+^ signals (IC_50_ of 1.3±0.3 μM) mediated by *Sm*.TRPM_PZQ_ (Fig 4F). When applied to intact worms ANT1 did not change worm motility, but at high concentrations (50μM) enhanced recovery of schistosome worms incubated in the continued presence of PZQ compared to worms treated with PZQ alone (Fig 4G).

**Fig 5:**
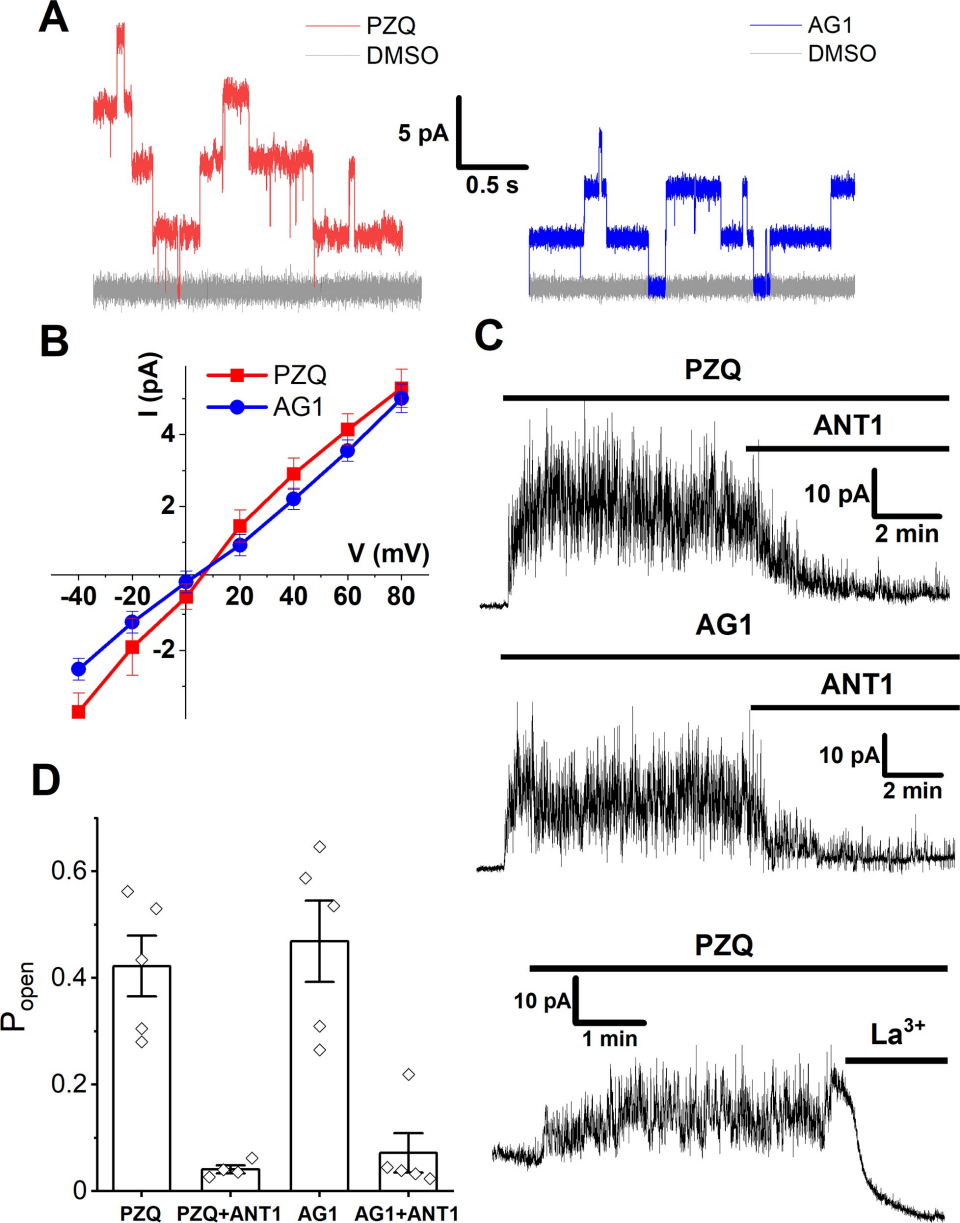
Electrophysiological analysis of new *Sm*.TRPM_PZQ_ chemotypes. (**A**) *Sm*.TRPM_PZQ_ channel fluctuations evoked by PZQ (red) or AG1 (blue) both applied at 10 μM compared to vehicle responses (grey, 0.1% DMSO). Recordings were made in Ca^2+^-free solution at a clamping potential of +60mV in an inside-out configuration. DMSO and the test drug (PZQ or AG1) were added sequentially to the same patch. (**B**) Current (I)-voltage (V) relationship of *Sm*.TRPM_PZQ_ activated with PZQ (10 μM, red) or AG1 (10 μM, blue). (**C**) Effect of ANT1 (top and middle, 50 μM) or La^3+^ (bottom, 10 mM) addition on *Sm*.TRPM_PZQ_ activity evoked by PZQ (10 μM) or AG1 (10 μM) measured in cell-free mode at +40mV using an inside-out configuration. (**D**) Measurements of single channel open probability (P_open_) under the indicated conditions. Welch’s t-test: PZQ vs ‘PZQ+ANT1’, p = 0.002; AG1 vs ‘AG1+ANT1’, p = 0.004.

To validate ANT1 using an orthogonal assay, ANT1 action versus PZQ and AG1 was assessed using an electrophysiological approach. Single channel recordings of *Sm*.TRPM_PZQ_ activity were made in Ca^2+^-free buffer following addition of either PZQ or AG1 (10 μM) to a cell-free patch in ‘inside-out’ recording mode. Both PZQ and AG1 evoked step-wise openings of *Sm*.TRPM_PZQ_ (Fig 5A) with channel activation defined by linear I-V relationship in response to both agonists (Fig 5B). The mean slope single-channel conductance for PZQ- and AG1- activated *Sm*.TRPM_PZQ_ was 76 ± 8 and 61 ± 4 pS respectively (mean ± SD). Addition of PZQ, or AG1, evoked sustained, non-desensitizing *Sm*.TRPM_PZQ_ currents, which were blocked by subsequent addition of ANT1 in a time-dependent manner (Fig 5C). Analysis of single channel open probability (P_open_) from these records demonstrated ANT1 decreased P_open_ at *Sm*.TRPM_PZQ_ channels activated by either agonist (Fig 5D). After ANT1 application, brief channel openings persisted in the presence of PZQ or AG1 that were not seen in the presence of PZQ after addition of the pore blocker La^3+^ [7]. This observation is again suggestive of a competitive interplay between ANT1 and the channel activators, PZQ or AG1, within the orthosteric binding pocket of *Sm*.TRPM_PZQ_.

In summary, these data reveal that AG1 behaves a novel activator of *Sm*.TRPM_PZQ_ and ANT1 as a blocker of PZQ (or AG1) action at *Sm*.TRPM_PZQ_. Both these ligands have structures different from PZQ, evidencing the druggability of *Sm*.TRPM_PZQ_ by novel chemotypes.

## Discussion

Here, we report successful miniaturization of a reporter assay (6μl assay volume) to monitor the activity of a parasitic flatworm ion channel, *Sm*.TRPM_PZQ_. This approach will enable further HTS campaigns to provide insight about the properties and regulation of this recently discovered flatworm ion channel that is targeted by PZQ [8,9]. Critically, the ability to screen diverse libraries provides opportunity to discover new chemotypes active at this channel complex. This is important as the structure-activity relationship of PZQ has long been known be ‘tight’ with even minor modifications to the ligand causing a marked decrease in activity [9]. This has frustrated efforts to rationally engineer PZQ derivatives with enhanced properties, or improved metabolic stability. Alternative chemotypes to PZQ are required, both to mitigate some of the deficiencies of this stalwart therapeutic, as well as to bolster the global drug development pipeline [4]. Our knowledge of the properties of the PZQ binding site in TRPM_PZQ_ derive from recent mutagenesis and modeling studies guided by analyses of responses to PZQ and related derivatives [9]. However, an expansive body of work on the human TRPM8 channel evidences accommodation of various chemotypes within a malleable transmembrane binding pocket [22,23], that overlaps with the binding site for PZQ characterized in *Sm*.TRPM_PZQ_ [9]. Indeed (*S*)-PZQ engages this binding pocket in hTRPM8 [24,25]. Such data justify screening for new chemotypes active at flatworm TRPM_PZQ_ channels.

For these reasons, we optimized methods to allow a pilot screen of ~16,000 compounds at *Sm*.TRPM_PZQ_. After titration and counter-screening, a single novel activator of *Sm*.TRPM_PZQ_−for simplicity referred to as AG1 –was identified and validated in an orthogonal assay via single channel recording. Application of AG1 to schistosome worms *ex vivo* also caused a rapid contraction (Fig 4D). Interestingly, AG1 is not a novel chemical entity, it was previously prioritized in an earlier phenotypic screen. Mansour *et al*. executed a screen of ~300,000 compounds against larval, juvenile, and adult schistosomes [26]. AG1 (LSHTM-1507) was one of seven hits prioritized from the phenotypic screening pipeline [26] all of which displayed activity against each of the three life cycle stages, a good cytotoxicity window and structural attractiveness for further derivatization [27]. A second ‘hit’ from the same screen (LSHTM-1945) is a closely related chemical structure, and a third compound (LSHTM-1956) was a derivative of a mammalian TRPC6 inhibitor [28]. AG1 was also identified as an anti-schistosomal compound in previous screens of the Pathogen Box executed by independent laboratories [29]. That a phenotypic screen of ~300,000 compounds [26], inherently blind to the mode of action of many of the screened ligands, should identify one (possibly more) *Sm*.TRPM_PZQ_ activators highlights an impressive convergence of phenotypic and target-based screening methods and underscores the relevance of TRPM_PZQ_ as a druggable target. These data support the execution of a larger target-based screen to identify *Sm*.TRPM_PZQ_ activators, especially given the low hit rate for agonist discovery observed from this foundational screening effort (1 from ~16,000 compounds).

Just like PZQ, AG1 was more active against adult worms than juveniles [26], and the activity reported in phenotypic assays (IC_50_ of ~1.6 μM, [26]) mirrors the activity range for *Sm*.TRPM_PZQ_ activation *in vitro* (1.3 μM, Fig 4B). However, AG1 represents a discrete chemotype from PZQ. It is a triazolopyridine derivative and a considerably more hydrophobic ligand (M_r_ = 373.4, XLogP3-AA = 5.5, TPSA = 48.6 Å^2^) compared with PZQ (M_r_ = 312.4, XLogP3-AA = 2.7, TPSA = 40.6 Å^2^). The higher hydrophobicity of AG1 may contribute to the slower kinetics of action on intact worms *ex vivo* (Fig 4D). PZQ derivatives previously shown to activate *Sm*.TRPM_PZQ_ displayed attributes tightly clustered around those of the parent ligand (average properties of agonists with EC_50_<10 μM, M_r_ = 323, average LogP = 2.3 [9]). However, the physiochemical properties of AG1 are not dissimilar from other classes of ligands that activate human TRPM8 (hTRPM8, [23]). It is therefore exciting to have discovered a novel agonist chemotype which activates *Sm*.TRPM_PZQ_ (Fig 4). Preliminary mutagenesis data with AG1 (Fig 4B), and the similar effects of ANT1 on AG1- and PZQ-evoked *Sm*.TRPM_PZQ_ activity (Fig 5C), suggest that AG1, like PZQ, functions as an orthosteric ligand occupying the transmembrane binding pocket. Further mutagenesis work will be needed to expand these observations and resolve the relative binding poises of the two drugs. This comparison may hold significance as binding site mutants that impair PZQ binding may not necessarily impair the interaction of AG1, or other channel activators. This is known for hTRPM8 ligands [9]—for example, the conservative replacement hTRPM8[R842K] inhibits PZQ activation of hTRPM8 but has no effect on activation by cooling agent WS-12. Similarly, hTRPM8[H845W] abolishes responses to icilin but not PZQ [9]. This could prove an important observation in the context of engaging TRPM_PZQ_ channels refractory to PZQ, for example in *Fasciola* species [9].

The screen also identified a larger number of potential *Sm*.TRPM_PZQ_ blockers (32 from 16,000 compounds), which require further investigation to define their mode of action. For example, do these ligands act as pore blockers or as binding site antagonists? The utility of *Sm*.TRPM_PZQ_ blockers (as opposed to *Sm*.TRPM_PZQ_ activators) as anthelmintics is unproven, however their study and optimization has merit in providing selective tools for understanding worm physiology and the role of *Sm*.TRPM_PZQ_ *in vivo*. Here, we validated the blocking activity of a single of these candidates (ANT1), which antagonized PZQ action in the Ca^2+^ reporter (Fig 4F), electrophysiological (Fig 5C and 5D) and a worm contraction assay (Fig 4G). ANT1, like PZQ, contains a piperazin-1-yl methanone substructure. This structural resemblance, and the dissimilar action of ANT1 and La^3+^ on PZQ-evoked responses (Fig 5C and 5D), suggests ANT1 also acts as an orthosteric ligand.

In conclusion, this work has optimized methods for executing a highly miniaturized, large scale small molecule screen against a parasitic flatworm ion channel targeted by PZQ. Discovery of new chemotypes using these methods will help decipher features of *Sm*.TRPM_PZQ_ ligands critical for anthelmintic efficacy, and provide a workflow for screening campaigns at other parasitic flatworm ion channels.

## Materials and methods

### Ethics statement

All animal experiments followed ethical regulations approved by the MCW IACUC committee (AUA00006079/6476).

### Reagents

Cell culture reagents were from Invitrogen. Lipofectamine 2000 was from ThermoFisher. Libraries were sourced as follows: LOPAC_1280_ (Sigma), the Pathogen Box (Medicines for Malaria), Scripps Drug Discovery Library (Scripps Institute). FLIPR Calcium 5 assay kits were from Molecular Devices. Additional supply of AG1 (3-(3,4-Dimethoxyphenyl)-6-(3-propan-2-ylphenyl)-[1,2,4]triazolo[4,3-a]pyridine; PubChem CID:122196572, C_23_H_23_N_3_O_2_) was sourced from Evotec and ANT1 (1-(9H-fluoren-9-yl)-4-(5-methyl-3-phenyl-1,2-oxazole-4-carbonyl)piperazine; PubChem CID:2813918, C_28_H_25_N_3_O_2_) was sourced from Maybridge. All other routinely used chemical reagents were purchased from Sigma.

### HTS screening workflow

HEK cells were transfected with cDNA (400μg/ml DNA) encoding *Sm*.TRPM_PZQ_ using a Maxcyte STX electroporation system and incubated overnight at 37°C. This system is uniquely capabable of large scale transfections providing one batch of cells ready for large scale HTS [30]. Briefly, the HEK cells were resuspended at 10^8^/ml in Maxcyte electroporation buffer. Plasmid DNA was added to the cells in buffer to achieve a concentration of 400 μg/ml from a stock of 5mg/ml (in TE buffer). This mixture was loaded into the appropriate cassette, inserted into the machine, and cells electroporated. Cells were then frozen to provide a single supply of transfected cells for all subsequent assays. On the day of the assays, cells were thawed in a 37°C waterbath, and resuspended in DMEM + 10% FBS and counted. The cells were pelleted by centrifugation at 170xg for 5 minutes. The media was aspirated, and the remaining pellet was resuspended in 1x HBSS with 20 mM HEPES and 1% DMSO to achieve 1.33x10^6^cells/ml. This cell suspension was then dispensed at 3 μl/well to the 1536 well plates (Greiner Bio One part 789072) at a density of 4,000 cells/well. Measurements of fluorescence intensity (λex = 470 nm, λem = 535 nm) were made using a FLIPR (Molecular Devices FLIPR Tetra). A read of basal fluorescence values was made (5s) prior to addition of compounds or controls (30 nl). In both ‘agonist’ and ‘antagonist mode’, the total assay volume was 6μl (1% DMSO). For the primary screen, each compound was tested at a single concentration in triplicate, and responses compared on a per plate basis by evaluating the percentage response of each compound versus the ‘high control’ (response to EC_100_ PZQ (10 μM) for the agonist screen, DMSO for the antagonist screen)) corrected for ‘low control’ (response to DMSO for the agonist screen, response to EC_80_ PZQ (500 nM) in the presence of DMSO or compounds (nominally 5 μM) for the antagonist screen). Ligand performance was categorized using an algorithm based on the activity of the entire dataset to define a ‘hit-cutoff’ parameter for both agonist-mode and antagonist-mode screening. Any compound with greater percent activation than this cutoff was assigned as ‘active’ prior to titration and counter-screening. Titration assays were executed using the same reagents, protocols and detection systems, done as 10-point concentration-response curves, performed in triplicate. Counter-screening was performed using untransfected HEK293 cells for comparison with results testing the same compounds effect on cells expressing *Sm*.TRPM_PZQ_, with ATP used as the positive control. For each compound, activity was plotted against compound concentration and data were fitted with a sigmoidal function. In order to be considered a hit at this stage a compound must achieve a EC_50_<5 μM for agonists and IC_50_ <5 μM for antagonists. All compounds selected for titration were subjected to LC-MS analysis to confirm mass and sample purity. ChemAxon (https://www.chemaxon.com) academic licensing was provided for the use of Instant JChem (ver. 15.10.12.0) to perform compound mining of the libraries utilized in this effort.

### Schistosome mobility assays

Adult schistosomes were harvested from the mesenteric vasculature of female Swiss Webster mice previously infected (~49 days earlier) with *S*. *mansoni* cercariae (NMRI strain) by the Schistosomiasis Resource Center at the Biomedical Research Institute (BRI, Rockville, MD). Harvested schistosomes were washed in RPMI 1640 supplemented with 5% heat inactivated FBS (Gibco), HEPES (25 mM) and penicillin-streptomycin (100 units/mL). After isolation, worms were incubated overnight (37°C/5% CO_2_) in vented petri dishes (100x25 mm). Movement assays were performed using male and female worms in six well dishes (~5 individual worms/3ml media per well). Video recordings of worm motility (4 frames/sec) were captured using a Zeiss Discovery v20 stereomicroscope coupled to a QiCAM 12-bit cooled color CCD camera controlled by Metamorph imaging software [31].

### Electrophysiology

For electrophysiological analysis, a HEK293 cell line stably expressing *Sm*.TRPM_PZQ_ [7] was generated using the Flp-In T-REx system (K650001, Thermofisher Scientific, Carlsbad, CA). Flp-In T-REx HEK293 cells were cultured in Dulbecco’s modified Eagle’s medium supplemented with 10% fetal bovine serum, penicillin (100 units/ml), streptomycin (100 μg/ml), L-glutamine (290 μg/ml), blasticidin (10 μg/ml), and hydromycin B (100 μg/ml). HEK293 cells were plated onto glass coverslips and *Sm*.TRPM_PZQ_ expression induced by addition of tetracycline (2 μg/ml) to the media 24–48 hours before recording. Prior to recording, coverslips were secured within a recording chamber of an Olympus BX51WI upright microscope. Cells were bathed in a solution containing 145 mM KCl, 10 mM HEPES, 1 mM EGTA (pH 7.2, 310–315 mOsm/kg with sucrose). The pipette solution contained 140 mM LiCl, 10 mM HEPES, 1 mM EGTA (pH 7.4, 280–285 mOsm/kg with sucrose). Patch pipettes were made of borosilicate glass (BF150-110-10, Sutter Instrument, Novato, CA) pulled on a vertical puller (Narishige, Amityville, NY, Model PC-10) with resistances 8–10 MΩ. After gigaseal formation, membrane patches were excised from cells and current recorded in voltage-clamp mode in ‘inside-out’ configuration using a MultiClamp 700B amplifier and Digidata 1440A digitizer (Molecular Devices, Sunnyvale, CA). Signals were filtered with an 8-pole Bessel low pass filter at 1 kHz, and sampled at 20 kHz. Open probability (P_open_) was assessed in single channel search mode using Clampfit 10 software. All recordings were done at room temperature. Data represent mean±standard error for ≥3 independent experiments.

## Supporting information

S1 Table Assay performance in titration assays. Assay performance measured across 12 plates for 368 compounds in titration assays in untransfected HEK cells (counterscreen) and HEK cells expressing *Sm*.TRPM_PZQ_.
(DOCX)

S2 Table *Sm*.TRPM_PZQ_ agonists. Data from 3 compounds prioritized after titration assays. Compound descriptors and screening data in agonist and antagonist modes in HEK cells, and HEK cells expressing *Sm*.TRPM_PZQ_ are shown.
(XLSX)

S3 Table *Sm*.TRPM_PZQ_ antagonists. Data from 32 compounds prioritized after titration assays. Compound descriptors and screening data in agonist and antagonist modes in HEK cells, and HEK cells expressing *Sm*.TRPM_PZQ_ are shown.
(XLSX)
