# Supplementary material for: Identification of novel modulators of a schistosome transient receptor potential channel targeted by praziquantel
Source: PLoS Negl Trop Dis. 2021 Nov 3;15(11):e0009898. doi: 10.1371/journal.pntd.0009898 (PMC8565742; doi:10.1371/journal.pntd.0009898)
Supplement: S1 Table — Assay performance measured across 12 plates for 368 compounds in titration assays in untransfected HEK cells (counterscreen) and HEK cells expressing Sm.TRPMPZQ. (DOCX) [file pntd.0009898.s001.docx]

**Table S1**

|  | **Z’** | **S:B** |
| --- | --- | --- |
| **HEK-*Sm*.TRPM_PZQ_ agonist** | 0.67±0.03 | 13.4±3.4 |
| **HEK-*Sm*.TRPM_PZQ_ antagonist** | 0.72±0.04 | 9.1±0.6 |
| **HEK agonist** | 0.53±0.04 | 3.8±0.4 |
| **HEK antagonist** | 0.50±0.03 | 5.5±0.5 |
